# Mapping the orbitofrontal cortex using temporal fluctuations in cerebral blood flow

**DOI:** 10.1002/brb3.2034

**Published:** 2021-01-13

**Authors:** Kalen J. Petersen, Manus J. Donahue, Daniel O. Claassen

**Affiliations:** ^1^ Department of Radiology and Radiological Sciences Vanderbilt University Medical Center Nashville TN USA; ^2^ Department of Neurology Vanderbilt University Medical Center Nashville TN USA; ^3^ Department of Psychiatry and Behavioral Sciences Vanderbilt University Medical Center Nashville TN USA

**Keywords:** functional MRI, magnetic resonance imaging, neuroanatomy, neuroimaging

## Abstract

**Introduction:**

The orbitofrontal cortex (OFC) is involved in diverse cognitive and behavioral processes including incentive valuation, decision‐making, and reinforcement learning. Anatomic and cytoarchitectonic studies divide the OFC along both medial‐lateral and rostral‐caudal axes. OFC regions diverge in structure and function, assessed in vivo using white matter tractography and blood oxygenation level‐dependent (BOLD) MRI, respectively. However, interpretation of *T*_2_*‐weighted BOLD is limited by susceptibility artifacts in the inferior frontal lobes, with the spatial pattern of these artifacts frequently assuming the geometry of OFC organization. Here, we utilize a novel perfusion‐weighted arterial spin labeling (ASL) functional connectivity approach, which is minimally susceptibility‐weighted, to test the hypothesis that OFC topology reflects correlated temporal hemodynamic activity.

**Methods:**

In healthy participants (*n* = 20; age = 29.5 ± 7.3), 3D ASL scans were acquired (TR/TE = 3,900/13 ms; spatial resolution = 3.8 mm isotropic). To evaluate reproducibility, follow‐up scanning on a separate day was performed on a participant subset (*n* = 8). ASL‐based connectivity was modeled for gray matter OFC voxels, and *k*‐means clustering (*k* = 2–8) applied to correlation statistics.

**Results:**

These approaches revealed both medial‐lateral and rostral‐caudal OFC divisions, confirming our hypothesis. Longitudinal reproducibility testing revealed 84% voxel clustering agreement between sessions for the *k* = 2 solution.

**Conclusion:**

To our knowledge, this constitutes the first in vivo cortical parcellation based on perfusion fluctuations. Our approach confirms functional OFC subdivisions predicted from anatomy using a less susceptibility‐sensitive method than the conventional approach.

## Graphical abstract

The orbitofrontal cortex (OFC) is critical to numerous cognitive and behavioral functions, and parcellation of this region has been proposed based on anatomy. However, its functional connectivity is poorly understood due to susceptibility artifacts in BOLD fMRI. Here we use arterial spin labeling and connectivity‐based clustering to show that the OFC reproducibly divides along medial‐lateral and rostral‐caudal axes.

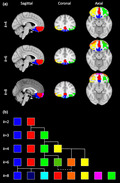

## 1 INTRODUCTION

The orbitofrontal cortex (OFC) occupies the ventral frontal lobe; it is central to sensory integration with reward circuitry, monitoring the values of environmental stimuli in the context of learning and decision‐making (Kringelbach, 2005). The OFC receives input from olfactory, gustatory, and somatosensory cortex (Rolls, 2002), and its position at the nexus of emotional processing and sensory integration enables it to mediate behavioral responses to stimuli. One of the OFC's major functions is to encode the perceived or expected value of reinforcers by linking sensory and limbic circuits (Schoenbaum et al., 2006). OFC pathology has been extensively implicated in behavioral dysregulation, especially in the context of addiction (Volkow & Fowler, 2000).

Evidence from a meta‐analysis of functional MRI (fMRI) studies suggests that the OFC is divisible into medial and lateral networks. These subdivisions serve different roles in decision‐making, with medial OFC attuned to rewarding stimuli and lateral OFC more responsive to punishing stimuli (Kringelbach, 2005). A rostral‐caudal gradient is also proposed, with caudal regions responding to simple primary reinforcers, such as food, and rostral regions responding to indirect secondary reinforcers, such as money (Sescousse et al., 2010).

These findings highlight the requirement for detailed mapping of OFC functional connectivity and internal divisions to understand reward and addiction circuitry. OFC parcellation is often reported from anatomical or histological evaluations in non‐human primate models, post‐mortem human brain dissection, or diffusion tensor imaging, which have enabled detailed structural mapping of the inferior frontal cortex. However, in vivo functional imaging, particularly in the medial‐caudal OFC, is not as well‐developed owing to signal dropout and distortion from magnetic field inhomogeneity induced by air‐tissue interfaces in the sinuses (Cordes et al., 2000). The signal‐to‐noise ratio in traditional BOLD fMRI sequences is diminished in the OFC compared to other frontal areas. Thus, novel methods less sensitive to magnetic susceptibility are needed to accurately measure OFC functional connectivity.

Arterial spin labeling (ASL) is a noninvasive MRI technique which is minimally susceptibility‐weighted and primarily cerebral blood flow (CBF)‐weighted. ASL reports on the rate of blood delivery to tissue (CBF; ml blood/100 g/min), closely correlated with the cerebral rate of glucose metabolism (Jueptner & Weiller, 1995). Due to shorter echo times in ASL (TE = 7–13 ms) relative to BOLD (TE = 25–45 ms), ASL is less susceptibility‐weighted and less vulnerable to OFC imaging artifacts.

While ASL has been successfully used to evaluate evoked hemodynamic activity (Donahue et al., 2009; Lu et al., 2004; Obata et al., 2004), it is also a promising albeit relatively novel approach for performing resting‐state functional connectivity analyses, as its signal source is specific to capillary rather than venous vasculature. ASL‐based functional connectivity has been shown to resemble brain resting‐state networks with fidelity comparable to BOLD functional connectivity (Jann et al., 2015; Li et al., 2018; Petersen et al., 2017), and can be applied to regions where BOLD connectivity is suboptimal (Dai et al., 2016; Munsch et al., 2020).

Here, we utilize this method to test the hypothesis that OFC parcellation from resting‐state ASL follows known structural subdivisions. If confirmed, this would provide further evidence supporting medial‐lateral and rostral‐caudal models, and motivate the use of ASL as a candidate technology in application studies of reward and addiction.

## 2 METHODS

### 2.1 Imaging

Healthy participants provided written, informed consent. This study was approved by the Institutional Review Board at Vanderbilt University Medical Center and accords with the Declaration of Helsinki. Scanning was performed using a 3‐Tesla Philips Ingenia scanner with body coil transmit and SENSitivity Encoding (SENSE) 32‐channel array reception. Participants were instructed to remain awake with eyes open; wakefulness was monitored before and after each scan.

20‐min resting‐state pseudo‐continuous ASL (pCASL) scans were optimized in preliminary work to maximize temporal signal‐to‐noise ratio while maintaining temporal resolution. These parameters were varied: labeling train duration, post‐labeling delay, use of background suppression, number of inversion pulses, readout direction, spatial resolution, and voxel size anisotropy. Subsequent pCASL images were acquired with the following parameters: TR/TE = 3,900/13 ms, post‐labeling delay = 1,800 ms, label duration = 1,800 ms, field‐of‐view = 304 × 304 × 95 mm, spatial resolution = 3.8 mm isotropic. Four‐pulse background suppression was utilized. The readout consisted of a 300 ms 3D Cartesian gradient and spin echo (GRASE) module with SENSE (in‐plane acceleration = 3, through‐plane = 2). A segmented 3D GRASE readout (Alsop et al., 2015) was not used, so as to obtain a higher temporal resolution and enable connectivity determination over a physiological frequency range (0.01–0.10 Hz). Representative pCASL images and temporal signal‐to‐noise ratio maps are shown in Figure S1.

3D *T*_1_‐weighted magnetization‐prepared rapid gradient‐echo (MPRAGE) scans (TR/TE = 8.9/4.6 ms) were acquired for OFC definition and co‐registration. To measure reproducibility, a subset of participants returned for an identical scan session on a separate day.

Image preprocessing, including motion correction and denoising, was performed using the FMRIB Software Library (FSL; Smith et al., 2004), all other image processing, analysis, and statistics utilized custom Matlab scripts (Mathworks). ASL images were motion‐corrected using MCFLIRT (Jenkinson et al., 2002). Surround subtraction was applied (Lu et al., 2006): each spin‐labeled image was subtracted from the mean of the preceding and following unlabeled images. This is preferable to simple paired subtraction as it reduces frame‐to‐frame variability and matches BOLD effects between control and label acquisitions. ASL data were smoothed using a full‐width‐at‐half‐maximum (FWHM) = 5 mm Gaussian kernel. Finally, component‐based noise correction (CompCor) was applied to denoise the images (Behzadi et al., 2007). Independent component analysis was performed on the ASL time series in a combined white matter‐cerebrospinal fluid nuisance ROI, and the top 5 components from this analysis were regressed out of all brain voxels to reduce the contribution from motion and physiological artifacts. ASL images were linearly registered to native *T*_1_‐weighted images using FLIRT. *T*_1_‐weighted images were nonlinearly warped to MNI space, and these transformations were applied to 4D ASL images to produce a 4‐mm MNI‐space ASL series.

**FIGURE 1:**
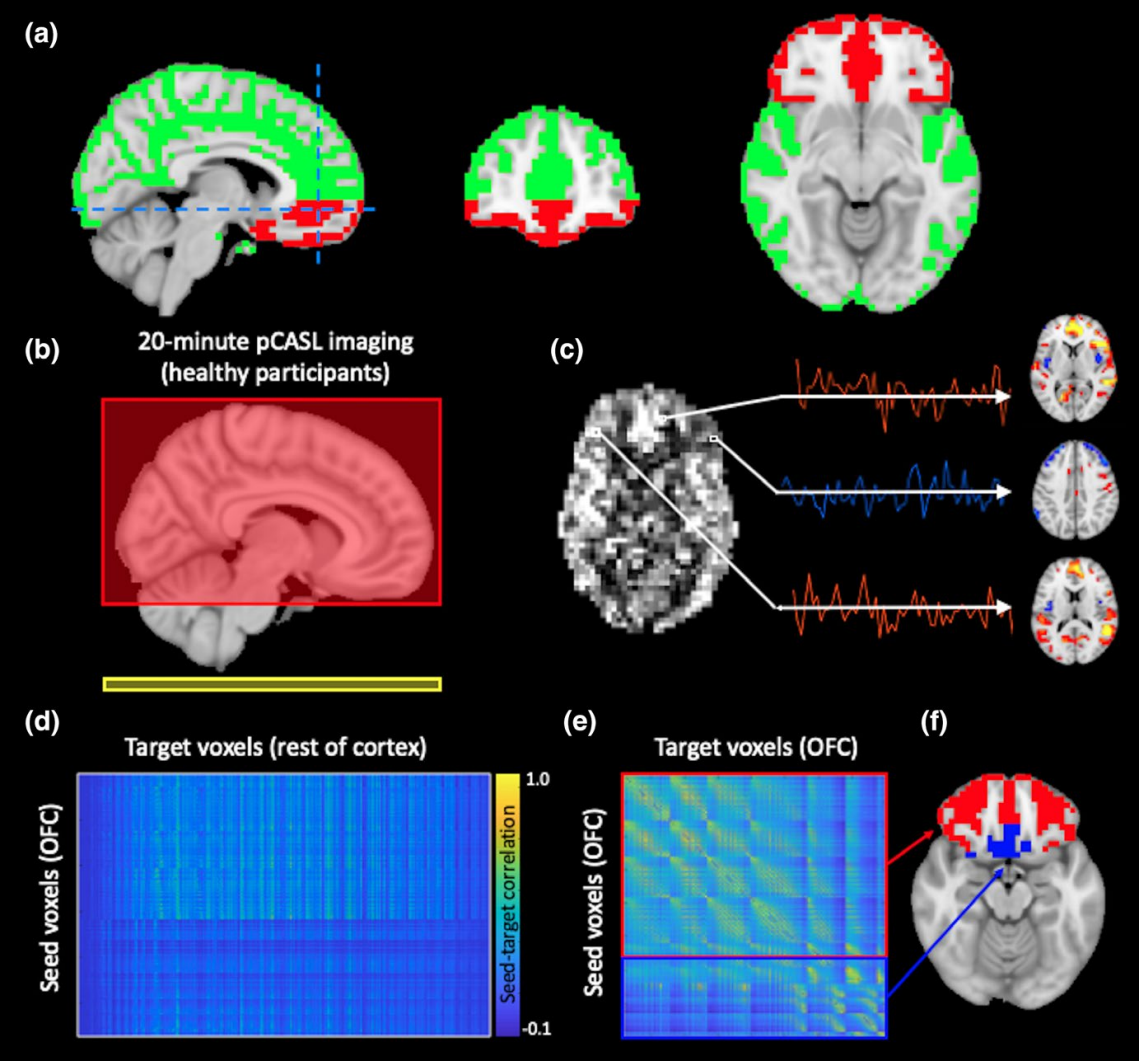
Study overview. (a) Orbitofrontal cortex (OFC, red) and whole‐brain gray matter (green) regions‐of‐interest were defined in 4‐mm MNI space. (b) Perfusion‐weighted pseudo‐continuous ASL (pCASL) MRI imaging was acquired in 20 healthy participants (labeling plane, yellow; image volume, red). (c) Dynamic perfusion time series were taken from each OFC voxel and used to generate functional connectivity matrices (d, e), which were used to parcellate the OFC. Both (d) extrinsic; OFC‐to‐whole brain) and (e) intrinsic (OFC‐to‐OFC) connectivity profiles were generated

An OFC mask was defined in MNI space, extending rostrally to the frontal pole, caudally to the anterior boundary of the insula at its separation from the temporal lobe, inferiorly to the lowest extent of the frontal lobe, and superiorly to the lowest slice of the corpus collosum genu (Figure 1a). This definition included broad regions of inferior prefrontal cortex, including those consistently termed OFC, Brodmann's areas (BA) 47 and 11 (sensu *lato*, including BA 12), and more peripheral areas (inferior portions of BA 10 and 25). The *T*_1_‐weighted MNI152 2‐mm brain atlas was down‐sampled to 4‐mm isotropic voxels to approximate the acquired spatial resolution of the ASL scans, then segmented into gray and white matter using FSL FAST; white matter voxels were excluded from the OFC owing to the blood arrival time in white matter (1.5–2 s) being on the order of the arterial blood *T*_1_ (1.6–1.8 s at 3T).

For connectivity analysis, a clustering‐based parcellation (CBP) method similar to (Kahnt et al., 2012) was employed. Functional connectivity analysis for OFC parcellation was performed using two complementary methods with mutually exclusive input data. First, parcellation was performed on the basis of *extrinsic* connectivity: OFC to non‐OFC gray matter. Second, parcellation was performed for *intrinsic* OFC connectivity: between voxels inside the anatomically‐defined OFC gray matter. Both methods may have relevance to future OFC connectivity studies, and therefore results from both approaches are presented, along with the consensus of the two methods.

### 2.2 Functional connectivity and parcellation

Extrinsic connectivity maps were created by calculating Pearson's correlations between time series in OFC and non‐OFC gray matter. An [(*m* − *n*) × *n*] connectivity matrix was calculated, where *m* is the total number of gray matter voxels and *n* is the number of OFC gray matter voxels. These matrices were averaged across all participants in MNI space. Hard clustering (*k*‐means) was then applied to the resulting matrix, so that OFC voxels were clustered based on their extrinsic connectivity. *k*‐means clustering with *k* = 2 was performed to decompose the OFC into spatial clusters to test our primary hypothesis. As an additional exploratory analysis, clustering with *k* = 3, *k* = 4, *k* = 6, and *k* = 8 was performed to identify further subdivisions.

Next, intrinsic OFC connectivity was determined using a similar approach. Each OFC voxel time series was used to model connectivity with all other OFC voxels, resulting in an [*n* × *n*] connectivity matrix. This matrix was then used as an input in *k*‐means clustering to parcellate the OFC in a process identical to that described above.

To assess whether cluster solutions were robust across participants, connectivity‐based parcellation was repeated by randomly reducing the cohort size by a factor of two and performing a separate connectivity analysis and clustering on each half‐group. Random bifurcation was performed with 100 repetitions. The spatial divergence of clusters from the two half‐cohorts was assessed using the variation of information (VI) metric (Meila, 2003), which is well‐established for c†omparison of cluster assignments between cohorts or runs (Kelly et al., 2010). Higher VI represents greater cluster instability, and increasing *k*‐number generally increases VI in a logarithmic fashion. For each value of *k*, mean VI was calculated to determine the effect of cluster number on the spatial stability of parcellations.

Whole‐brain connectivity maps for both clusters from the *k* = 2 solution were determined by calculating the mean connectivity of all voxels contributing to that cluster. To describe how these connectivity profiles differ between clusters, the resulting single‐subject *z*‐maps were used as inputs into a one‐sample permutation test in FSL Randomize with 5,000 permutations and threshold‐free cluster enhancement (Smith & Nichols, 2009) to produce unique group‐level connectivity maps. The resulting group‐level *t*‐statistics were then subject to thresholding at *z ≥ *2.3, corresponding to a one‐sided *p* = .01.

### 2.3 Longitudinal reproducibility

Follow‐up imaging was preprocessed and extrinsic OFC connectivity was calculated using the method above. This group was used to assess the longitudinal reproducibility of clustering solutions based on overlap with the initial parcellation. Reproducibility was defined as the percentage of OFC voxels assigned to the same cluster between the initial and follow‐up parcellations.

## 3 RESULTS

### 3.1 Imaging

Figure 1 provides an overview of the acquisition and analysis. 20 healthy persons (age = 29.5 ± 7.3 years, sex = 10M/10F) participated. A subset (*n* = 8; age = 27.6 ± 5.0 years, sex = 4M/4F) returned for an identical follow‐up protocol on a separate date (mean gap = 30.1 days).

### 3.2 Functional connectivity and parcellation

**FIGURE 2:**
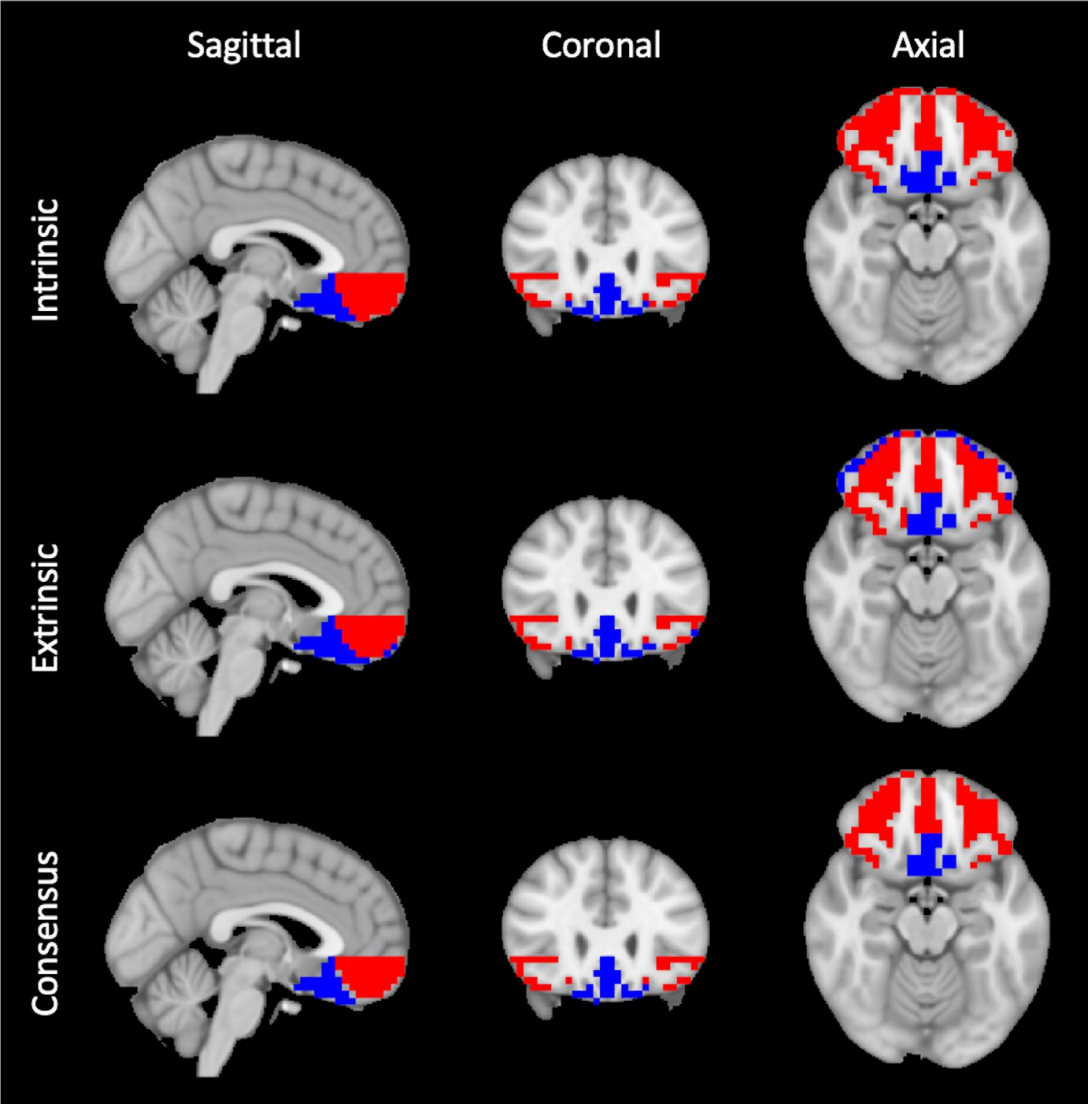
*k* = 2 cluster solutions. *k*‐means clustering reveals two primary OFC components: a medial‐caudal cluster (blue) and a lateral‐rostral cluster (red). Results from intrinsic connectivity (OFC‐to‐OFC), extrinsic connectivity (OFC‐to‐gray matter), and the consensus of the two approaches are shown

*k* = 2 clustering from extrinsic and intrinsic connectivity identified similar OFC subdivisions: A medial‐caudal OFC cluster (blue) approximating posterior BA 11 (gyrus rectus) and BA 25, and a lateral‐rostral cluster (red) including BA 47, the inferior‐most parts of BA 10, and anterior BA 11 (gyrus rectus; Figure 2).

**FIGURE 3:**
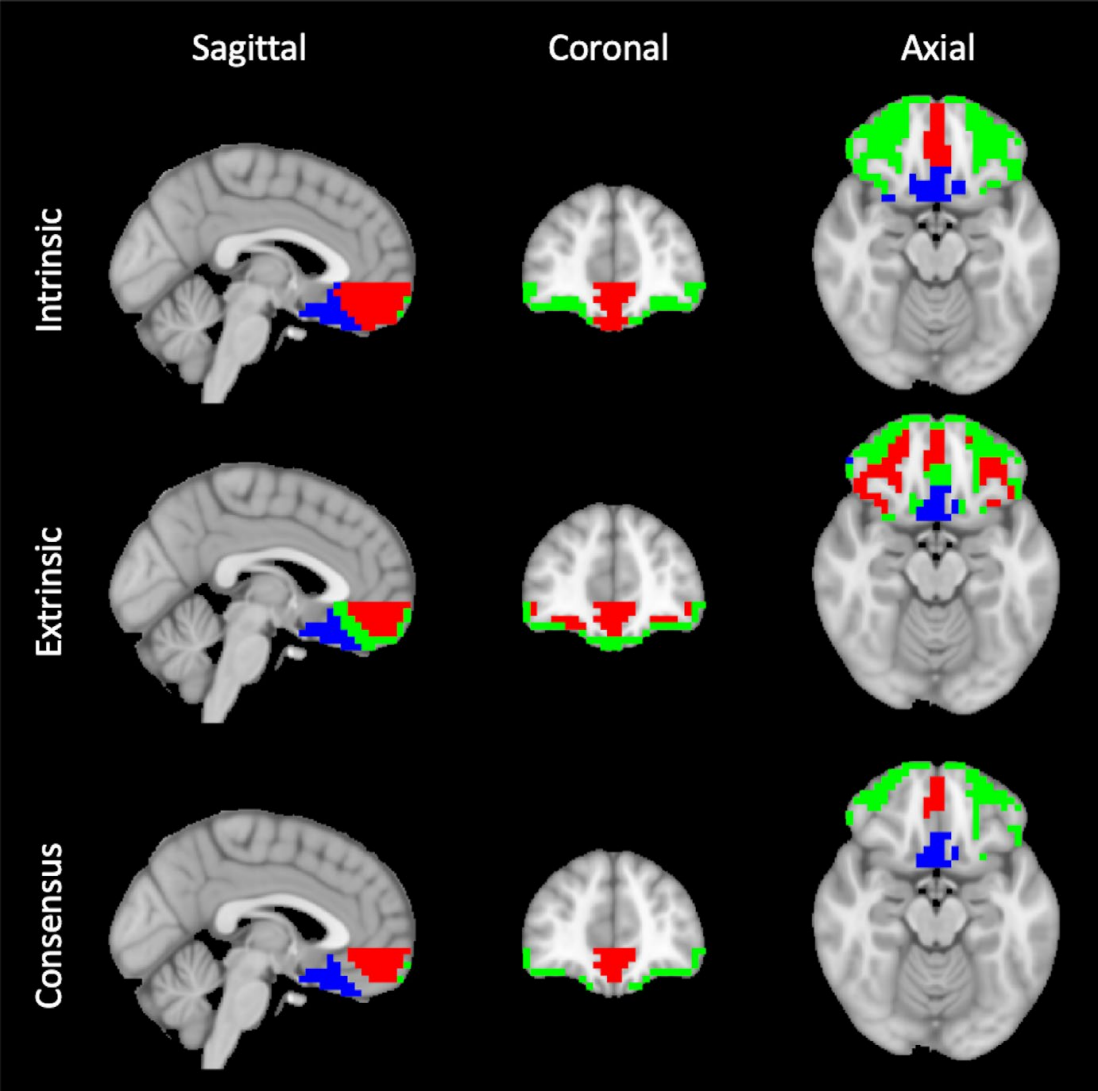
*k* = 3 cluster solutions. *k*‐means clustering reveals three OFC components: a medial‐caudal cluster (blue), a medial cluster in the gyrus rectus (red), and a lateral‐anterior cluster (green). Intermediate regions differed in cluster assignment between extrinsic and intrinsic approaches. Results from intrinsic connectivity, extrinsic connectivity, and consensus are shown

*k* = 3 extrinsic and intrinsic connectivity (Figure 3) both identified a distinct medial‐caudal cluster corresponding to BA 25 (blue), a medial cluster in the anterior gyrus rectus/BA 11 (red), and a lateral cluster including both left and right BA 10 (green). The extrinsic and intrinsic solutions differently assigned a bilateral region approximating BA 47, linked with the medial‐caudal zone in intrinsic analysis and with BA 11 in extrinsic analysis.

**FIGURE 4:**
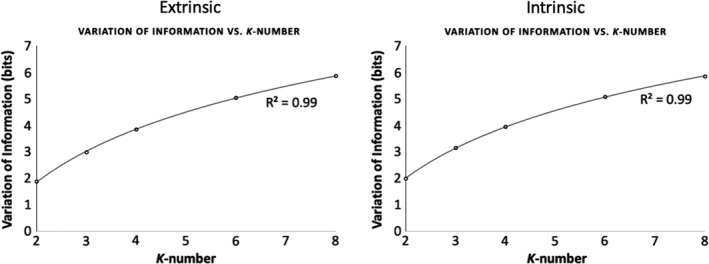
Variation of information increases with increasing cluster number. The variation of information (VI) metric was used to assess cluster instability between *k* = 2 and *k* = 8. A best‐fit curve for logarithmic growth is shown (*R*‐squared = .99 for both). VI is a metric of the dissimilarity between two clustering solutions, which increases with increasing complexity (parcellation entropy). Lower *k*‐number solutions are more repeatable, though less complex and detailed. These results were derived by performing 100 random splits of the participant cohort (*n* = 20) into two groups of 10, and performing *k*‐means clustering at all values of *k* for each split. VI values were then averaged across 100 splits

With increasing *k*‐number, the mean VI of cluster solutions increased in a logarithmic manner for both extrinsic and intrinsic connectivity, indicating increasing instability of more complex solutions, ranging from VI = 1.87 bits for *k* = 2 to VI = 5.86 bits for *k* = 8 (Figure 4).

**FIGURE 5:**
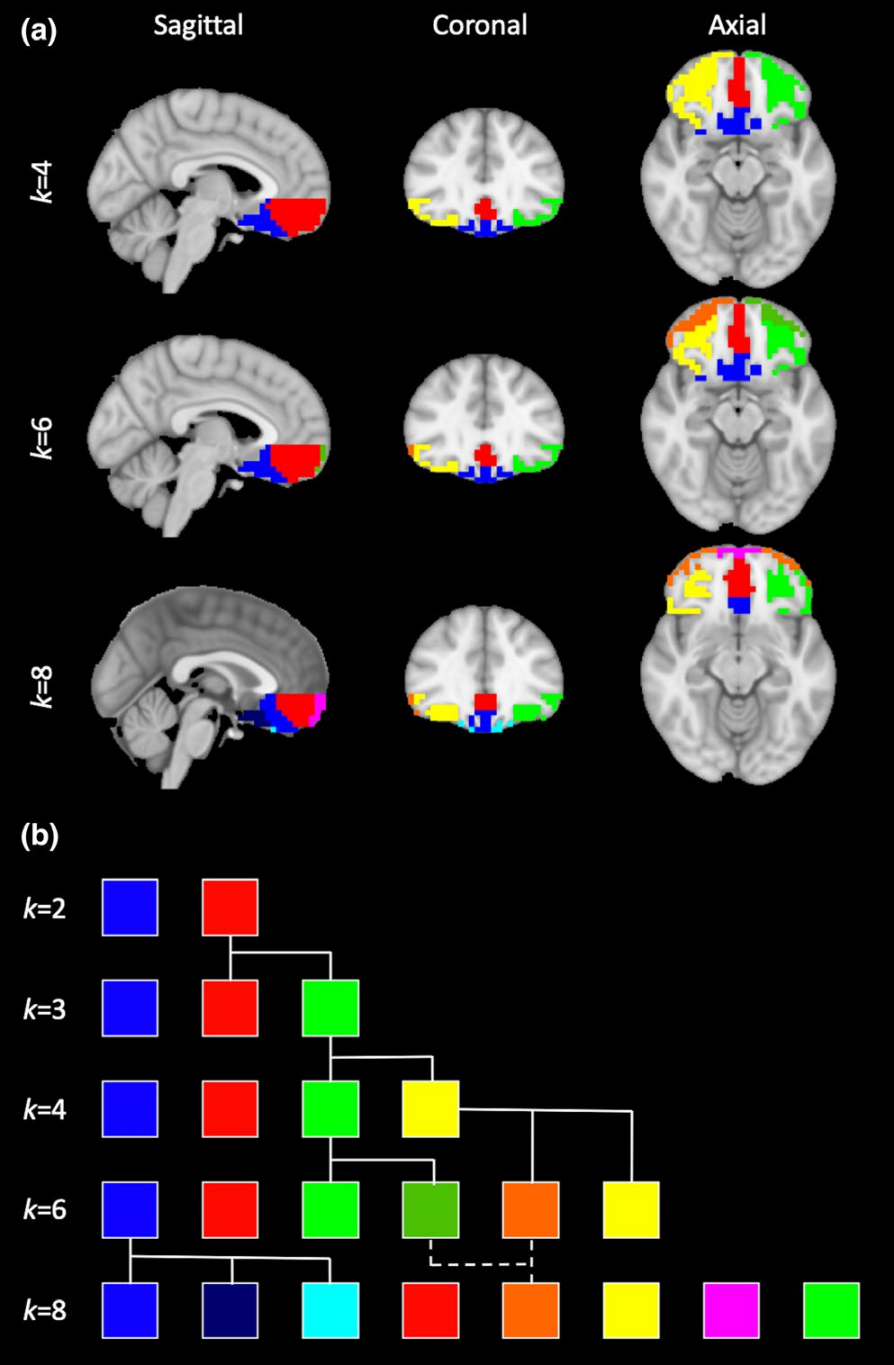
Higher‐order cluster solutions. (a) *k*‐means clustering for *k* = 4, *k* = 6, and *k* = 8 reveal a roughly hierarchical breakdown of OFC subregions. At *k > *3, intrinsic connectivity produced more spatially coherent clusters than extrinsic connectivity, and is presented here. (b) A hierarchical tree representing proposed relationships among clusters for increasing *k*. Branching lines represent proposed splits; a dashed line between *k = 6* and *k* = 8 corresponds to an apparent merge

Figure 5 summarizes the intrinsic clusters for *k* = 4, *k* = 6, and *k* = 8. The *k* = 4 solution closely matched the *k = 3* solution, except that the lateral‐rostral cluster was divided between left and right lobes (green, yellow). The *k = *6 solution further subdivided both left and right subregions into rostral and caudal sections (orange, dark green), while preserving the two medial clusters apparent at *k = *4 (red, blue). Finally, *k = *8 subdivided both medial and lateral clusters still further. The medial‐caudal cluster was split into 3 subregions (blue, dark blue, light blue), while rostral OFC was grouped into new medial and lateral regions (orange, magenta); the left and rightmost clusters remained separated (green, yellow).

**FIGURE 6:**
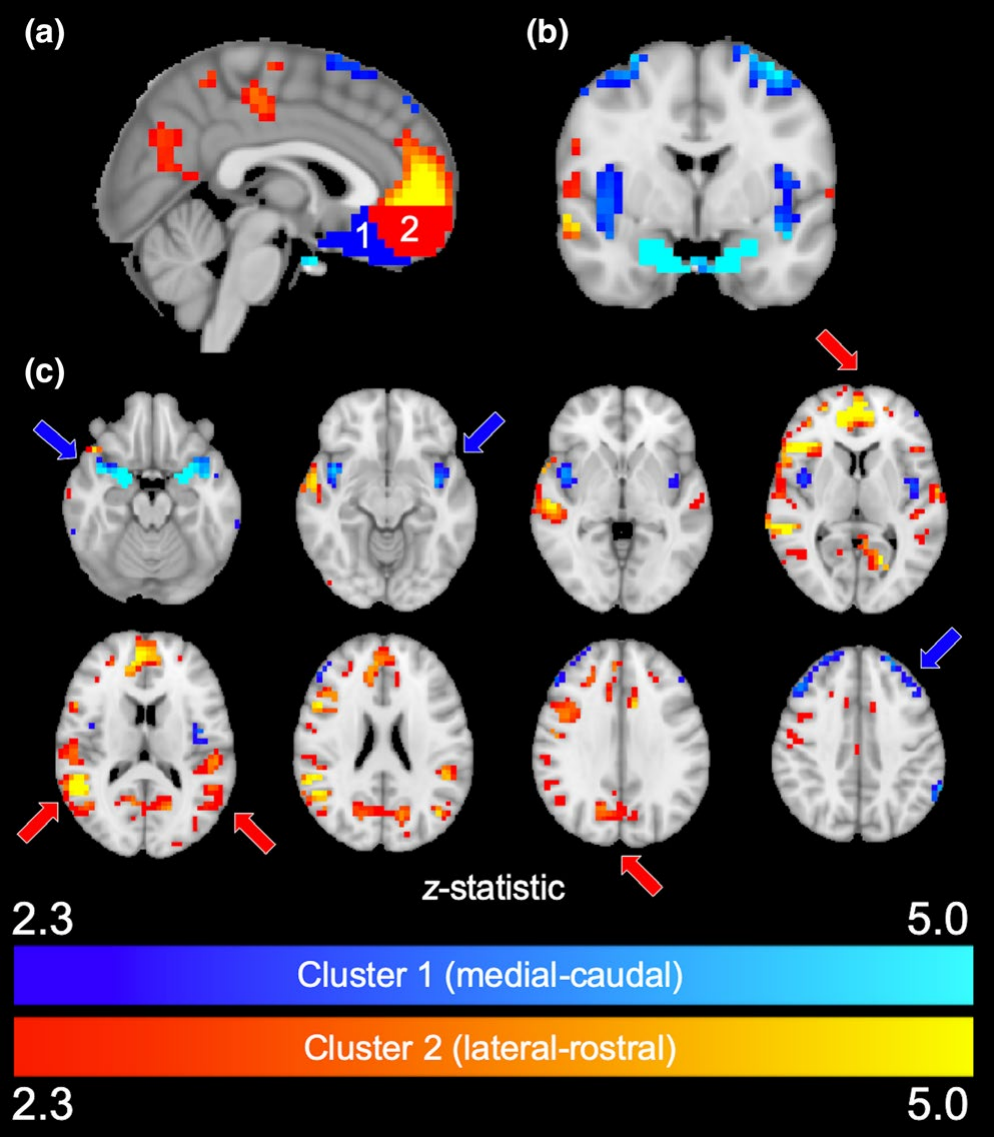
Connectivity of primary clusters. (a) Sagittal view of *k* = 2 clusters and their corresponding whole‐brain connectivity profiles. Regions uniquely connected with the medial‐caudal cluster (red) are represented in red–yellow. Regions connected with the lateral‐rostral cluster (blue) are represented in blue‐light blue. (b) Coronal view of connectivity maps. (c) Axial slices of connectivity maps. The medial‐caudal cluster shows strong connectivity with dorsolateral prefrontal cortex, amygdala, and insula (blue arrows), while the lateral‐anterior cluster is most connected with ventromedial prefrontal cortex, posterior cingulate gyrus/precuneus, and bilateral parietal cortex (red arrows)

To determine what cortical gray matter regions are uniquely connected with each primary OFC cluster, connectivity profiles of medial‐caudal and lateral‐rostral regions were statistically compared using permutation testing. Non‐OFC voxels with strong connectivity (*z ≥ *2.3) unique to either cluster are shown in Figure 6. The lateral‐rostral cluster (red) was most connected with the medial prefrontal cortex, posterior cingulate gyrus/precuneus, inferior parietal lobe, and temporal cortex. The medial‐caudal cluster (blue) was most linked with the bilateral insula and amygdala.

### 3.3 Longitudinal reproducibility

**FIGURE 7:**
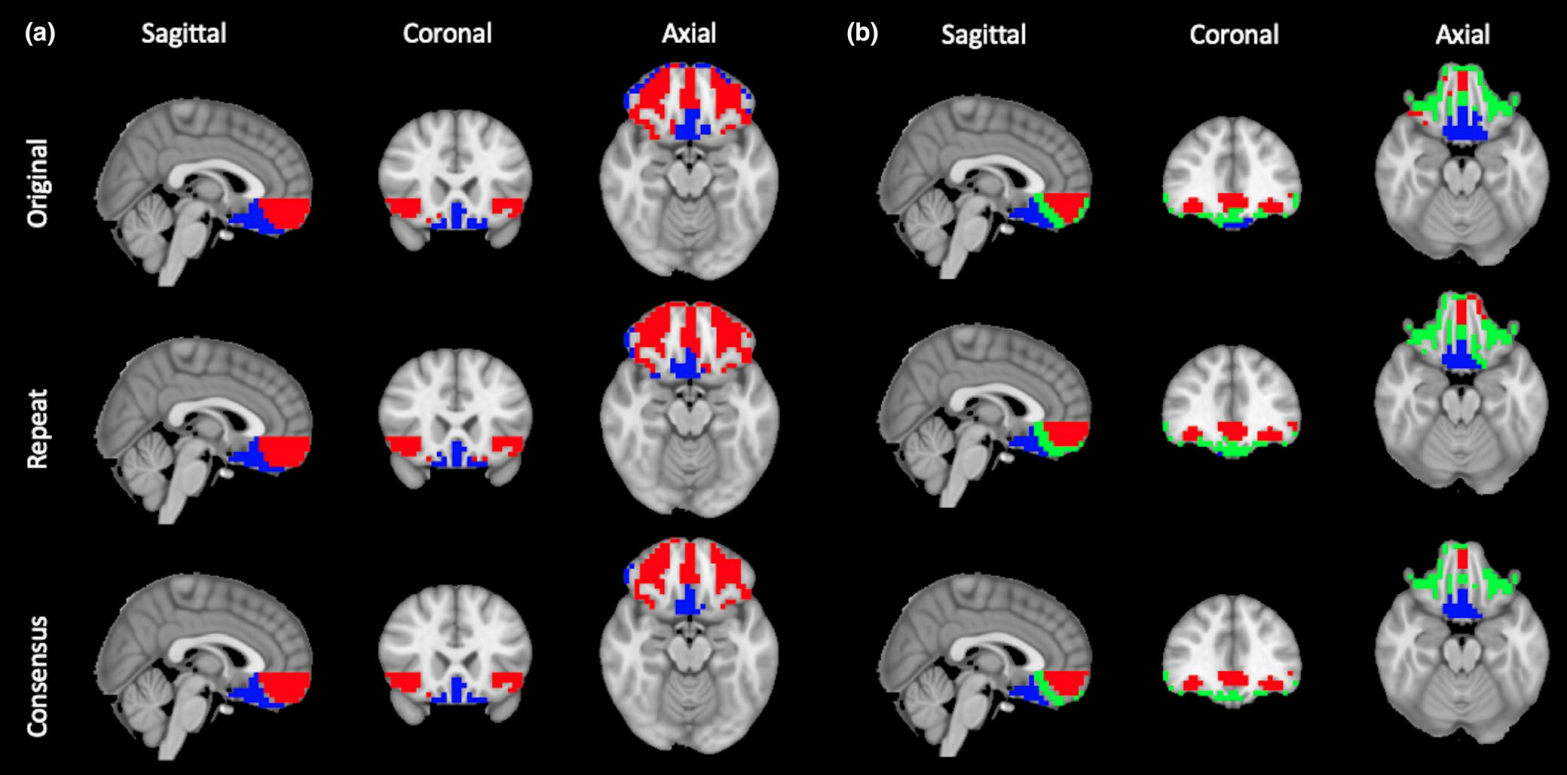
Longitudinal reproducibility of parcellation. (a) *k* = 2 cluster solutions derived from original scans and reproducibility follow‐ups show a high degree of overlap, as seen in the consensus panel. (b) *k* = 3 cluster solutions also show consistency across time, even with a reduced participant cohort

Reproducibility was determined by repeating the imaging procedure with a subset of participants on a separate day. Connectivity was recomputed as with the original images, and clusters were calculated for *k* = 2 and *k* = 3. For these solutions, respectively, there was 84% and 68% voxel agreement between the clusters derived from the original and repeated measurements (Figure 7).

## 4 DISCUSSION

The OFC has been mapped on the basis of microanatomy (Mackey & Petrides, 2009; Uylings et al., 2010), diffusion tensor imaging (DTI)‐based structural connectivity (Klein et al., 2007), and primate studies, both behavioral and anatomical (Noonan et al., 2010). However, existing human atlases do not account for functional connectivity between the OFC and large‐scale brain networks, omitting relevant data on extended and second‐order neural connections. BOLD fMRI experiments have begun to address this gap using both resting‐state (Kahnt et al., 2012) and task‐based paradigms (Zald et al., 2014); however, the potential for confounding magnetic susceptibility effects in inferior bifrontal lobes argues for a complementary approach using alternative imaging modalities to provide additional support for existing findings.

Therefore, we attempted to parcellate human OFC based on resting‐state fluctuations in cerebral blood flow, which are generally well‐correlated with the cerebral metabolic rate of glucose consumption and neuronal activity (Jueptner & Weiller, 1995). ASL is a logical candidate technology for future examination of the orbital frontal lobe, as it is less susceptibility‐weighted due to shorter echo times than typical gradient‐echo BOLD sequences.

Cytoarchitectonic studies support the proposition that OFC microanatomy varies along two axes: medial‐lateral and rostral‐caudal (Barbas & Pandya, 1989; Morecraft et al., 1992). Lateral BA 47 has larger neural cell bodies, more differentiated layers III and V, and a more granular layer IV than medial BA 11. Immunostaining also distinguishes BA 47 and 11 on the basis of reduced layer III neurofilaments and decreased layer II parvalbumin in the latter. Within BA 47, cortical thickness also decreases from lateral to medial (Uylings et al., 2010). Our results are generally consistent with these findings: all parcellations evinced clear medial‐lateral distinctions (Figures 2, 3 and 5).

Microanatomy also varies in the rostral‐to‐caudal direction: layer IV becomes increasingly dysgranular and gradually disappears, while layer V increases in prominence. This represents a transition from fully granular BA 10 to agranular BA 25, which border BA 47/11 rostrally and caudally. Likewise, BA 11 (gyrus rectus) undergoes a rostral‐caudal transition in which sublayer Va cells increase in size and sublayers Va and Vb become more distinct (Uylings et al., 2010). The same rostral‐caudal differentiation is apparent in our clustering results from *k* = 2 through *k* = 8 (Figures 2, 3 and 5).

Structural connectivity from both diffusion MRI‐based tractography and post‐mortem tract tracing support this view. Sensory pathways preferentially connect with lateral OFC, including later stages of visual processing (Barbas, 1988), while the medial OFC has unique and specific connections with the anterior cingulate (Cavada et al., 2000; Morecraft et al., 1992). Major human white matter pathways differentially connect with medial and lateral OFC: extreme capsule nerve tracts target lateral OFC, the uncinate fasciculus sends more projections to the central OFC, and projections from the amygdala target medial OFC (Croxson et al., 2005).

In the macaque, lateral OFC neurons project more strongly to areas within lateral OFC itself than to medial OFC; conversely, medial OFC neurons send more projections to each other than to lateral OFC (Carmichael & Price, 1996). This general agreement between intrinsic and extrinsic *structural* connectivity is paralleled by our finding that intrinsic and extrinsic *functional* connectivity are well‐matched (Figures 2 and 3).

Finally, limited functional imaging also supports the medial‐lateral hypothesis. In a meta‐analysis of task fMRI (Zald et al., 2014), co‐activation coordinates reported in BrainMap (Laird et al., 2005) were used to examine connectivity. Zald *et al*. found that lateral OFC is connected with prefrontal language and memory‐associated areas, while medial OFC is connected with autonomic and limbic regions. Loss‐of‐function studies indicate that this division is more than anatomical: lateral OFC lesions produce impaired reversal learning, causing primates to persist in responding to previously rewarded but currently unrewarded stimuli. Medial lesions, by contrast, impair reinforcement learning, such that the initial development of reward‐behavior associations is reduced (Iversen & Mishkin, 1970). Medial and lateral OFC may also have different roles in monitoring the values of environmental cues. Medial OFC is thought to respond primarily to rewarding stimuli and the lateral OFC to aversive stimuli (O'Doherty et al., 2001).

However, the above studies defined medial and lateral ROIs *a priori* on an anatomical basis. In contrast, the connectivity‐based parcellation (CBP) used here is a data‐driven approach to test hypotheses without anatomical assumptions. CBP using resting‐state fMRI data is an established technique to identify discrete cortical zones based on connectivity profiles (Eickhoff et al., 2015; Kim et al., 2010). Unsupervised clustering has defined whole‐brain networks (Cohen et al., 2008) or partitioned specific brain regions (Cauda et al., 2012; Deen et al., 2011; Kelly et al., 2010; Kim et al., 2010; Mishra et al., 2014; Zhang & Chiang‐shan, 2012). However, to our knowledge this is the first study to apply this approach to analysis of ASL‐based functional connectivity. Rather than cognitive testing or task‐based functional MRI, we instead utilized resting‐state blood flow fluctuations in healthy participants to describe OFC functional anatomy.

One previous study used CBP from resting‐state BOLD to divide the OFC, also revealing medial‐lateral bifurcation (Kahnt et al., 2012). Methodological differences present some challenges when comparing the two studies; for instance, Kahnt *et al*. utilized extrinsic connectivity, while our approach incorporated intrinsic and extrinsic connectivity. Our method is also less affected by magnetic susceptibility than *T*_2_*‐weighted gradient‐echo BOLD sequences. Nonetheless, the results seen here are broadly consistent with Kahnt *et al*., especially with regards to repeatable identification of medial and lateral networks. Medial clusters from our *k* = 6 solution (Figure 5, red, blue) approximate their *k* = 6 clusters 1 and 2, while our lateral clusters (green, orange, yellow) roughly correspond to their clusters 3–6. With regards to whole‐brain connectivity results, both studies detected connectivity between OFC and prefrontal cortex, insula, posterior cingulate, inferior parietal lobe, and temporal cortex (Figure 6).

In our study, extrinsic and intrinsic connectivity yielded congruent results, indicating that the internal OFC organization reflects common network participation throughout the brain. Both datasets identified clusters corresponding to medial‐caudal and lateral‐rostral zones, providing further evidence that the OFC is functionally divided along two primary axes. It is also noteworthy that OFC parcellations are mostly symmetrical, with medial‐lateral distinctions predominating over left‐right ones. This corresponds to microstructure, which also exhibits symmetric lateral‐to‐medial patterns (Uylings et al., 2010) and to structural connectivity, which follows the same arrangement (Croxson et al., 2005).

While these results are consistent with existing OFC connectivity data, it is important to consider alternative explanations. It is possible that delayed arterial arrival time between clusters might explain our CBP results. However, examination of the mean time‐series from the medial‐caudal and lateral‐rostral clusters did not reflect a simple time‐shifted relationship, suggesting that they were not distinguished primarily by offset blood arrival. We also quantified variance in cerebral blood flow, finding it to be significantly greater in the medial‐caudal cluster (*p* = .008; Figure S2), suggesting that greater temporal deviation in metabolic activity may play a role. Thus, we conclude that while vascular differences between OFC subregions exist, functional brain activity is likely the greatest contributor to parcellation.

These results should be contextualized with some limitations. First, the experimental design was intended to study healthy OFC; abnormal variations in hematocrit or baseline perfusion could produce spurious connectivity results in neurological disease (Donahue et al., 2012; Juttukonda et al., 2017). Second, ASL samples brain activity at longer intervals than standard BOLD sequences. The limited time resolution (TR ~ 7–8 s) means that ASL does not effectively capture some higher frequencies included in resting‐state BOLD. However, ASL connectivity depends on ultra‐low frequencies (<0.01 Hz) excluded from BOLD by baseline drift correction. Such correction is unnecessary in ASL, as control‐label subtraction inherently compensates for gradual instrument drift; therefore, ASL can examine connectivity over time scales inaccessible to other methods (Viviani et al., 2011). Capturing this low‐frequency regime requires a very long acquisition (~20 min), though ASL is noninvasive and well‐tolerated even by participants with neurological disorders. Alternative approaches such as electroencephalography might better capture the full range of timescales, though at the expense of spatial precision.

## 5 CONCLUSION

We utilized a data‐driven approach to show that dynamic cerebral blood flow reproducibly parcellates brain regions according to functional connectivity. The OFC divides along both medial‐lateral and rostral‐caudal axes, corresponding to previous evidence from both human imaging and animal models. Future ASL‐based connectivity studies may be relevant for interrogating OFC functional organization.

## CONFLICT OF INTEREST

The authors declare no relevant conflicts of interest.

## AUTHOR CONTRIBUTION

KJP, MJD, and DOC designed the current study. KJP and MJD recruited healthy controls and performed MRI scanning. KJP analyzed the data and wrote the manuscript. MJD and DOC helped to revise the paper. All authors read and approved the final manuscript.

### Peer Review

The peer review history for this article is available at https://publons.com/publon/10.1002/brb3.2034.

## Supporting information

Fig S1

Fig S2

## Data Availability

All data and code utilized in preparation of this manuscript will be made available upon request to the corresponding author.
