# Supplementary figures and images for: Mapping the orbitofrontal cortex using temporal fluctuations in cerebral blood flow
Source: Brain Behav. 2021 Jan 13;11(3):e02034. doi: 10.1002/brb3.2034 (PMC7994685; doi:10.1002/brb3.2034)

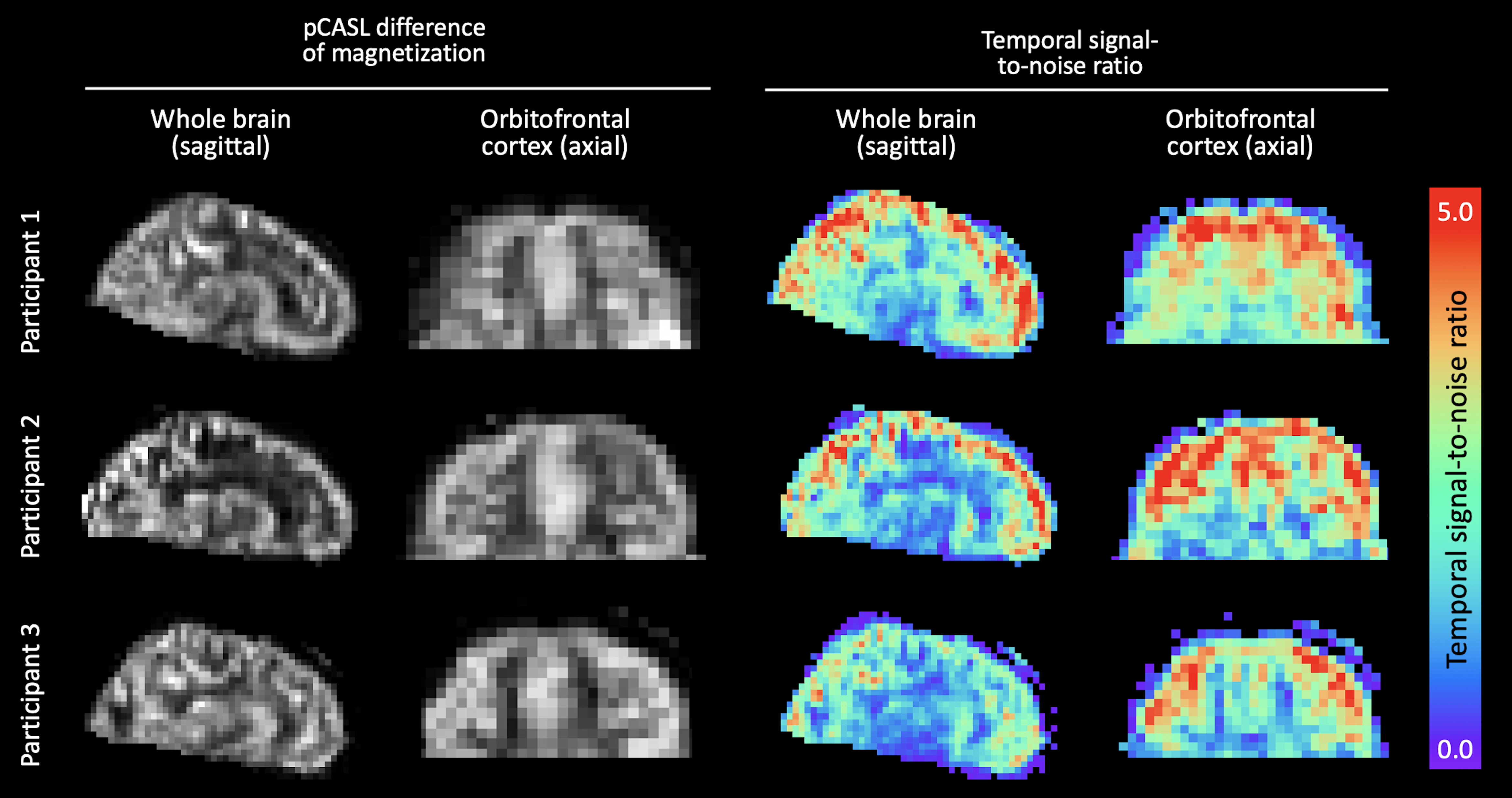

Supplement: Supplementary file 1 — Fig S1 [file BRB3-11-e02034-s002.png]

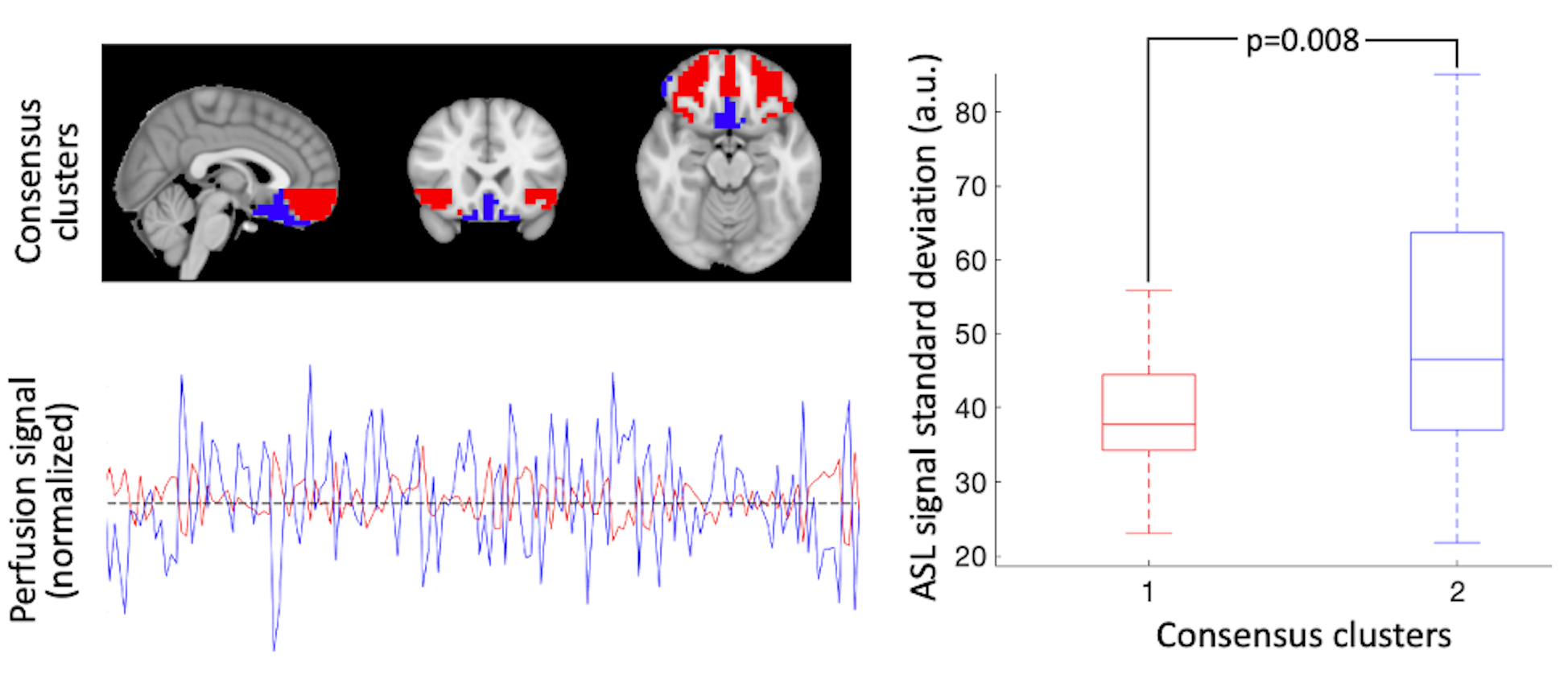

Supplement: Supplementary file 2 — Fig S2 [file BRB3-11-e02034-s001.png]
